# Influence of homophone processing during auditory language comprehension on executive control processes: A dual-task paradigm

**DOI:** 10.1371/journal.pone.0254237

**Published:** 2021-07-15

**Authors:** Samuel El Bouzaïdi Tiali, Elsa Spinelli, Fanny Meunier, Richard Palluel-Germain, Marcela Perrone-Bertolotti

**Affiliations:** 1 Université Grenoble Alpes, CNRS, LPNC UMR 5105, Grenoble, France; 2 Univ. Côte d’Azur, CNRS, BCL, Nice, France; 3 Institut Universitaire de France (IUF), Paris, France; San Diego State University, UNITED STATES

## Abstract

In the present preregistered study, we evaluated the possibility of a shared cognitive mechanism during verbal and non-verbal tasks and therefore the implication of domain-general cognitive control during language comprehension. We hypothesized that a behavioral cost will be observed during a dual-task including both verbal and non-verbal difficult processing. Specifically, to test this claim, we designed a dual-task paradigm involving: an auditory language comprehension task (sentence comprehension) and a non-verbal Flanker task (including congruent and incongruent trials). We manipulated sentence ambiguity and evaluated if the ambiguity effect modified behavioral performances in the non-verbal Flanker task. Under the assumption that ambiguous sentences induce a more difficult process than unambiguous sentences, we expected non-verbal flanker task performances to be impaired only when a simultaneous difficult language processing is performed. This would be specifically reflected by a performance cost during incongruent Flanker items only during ambiguous sentence presentation. Conversely, we observed a facilitatory effect for the incongruent Flanker items during ambiguous sentence suggesting better non-verbal inhibitory performances when an ambiguous sentence was simultaneously processed. Exploratory data analysis suggests that this effect is not only related to a more difficult language processing but also to the previous (*n-1*) Flanker item. Indeed, results showed that incongruent *n-1* Flanker items led to a facilitation of the incongruent synchronized Flanker items only when ambiguous sentences were conjointly presented. This result, even if it needs to be corroborated in future studies, suggests that the recruitment of executive control mechanisms facilitates subsequent executive control implication during difficult language processing. The present study suggests a common executive control mechanism during difficult verbal and non-verbal tasks.

## Introduction

A controversial question in the literature is whether language comprehension involves cognitive mechanisms specific for decoding linguistic information (i.e., domain-specific) or whether language comprehension implies more domain-general cognitive resources. Several authors suggested that, in all circumstances, language comprehension is performed by language-specific mechanisms only [1–4]. Other authors argue for a more domain-general account of language comprehension in which linguistic information processing could be shared with a larger machinery that encompasses other cognitive functions such as executive control (e.g., [5, 6]). In line with this approach, neuroimaging studies showed the involvement not only of the canonical language brain network (i.e. typical brain regions involved in language processing including the inferior frontal gyrus and the superior temporal cortex [5]), but also others brain networks such as the multiple demand network (see e.g. [7–10]). Some authors also suggest that executive resources are not critical for language processing, but their implication depends on the situation demands. In line with this last proposition, the *Good-Enough* approach [11] suggested that cognitive processes at work during language comprehension would depend on the situation requirements. Indeed, data from this approach showed that in most daily life situations only a heuristic analysis is performed as little information is sufficient to have a general idea of what has been said and to be able to communicate. Nevertheless, when the situation requires a deep understanding or when the speech is physically or linguistically degraded [12]; or when the linguistic information is complex [13] or ambiguous [14], then a domain-general mechanism is suggested to be involved and it allows a deeper and controlled analysis.

In this debate, the relationship between language processing and executive control has been mostly studied using neuroimaging methods showing an overlap of brain regions recruited during both executive control and language processing tasks [15–18]. January, Trueswell, and Thompson-Schill [19] showed such an overlap in an fMRI study between bold activity in the posterior left inferior frontal gyrus (LIFG) during high conflict sentence comprehension and incongruent Stroop trials. This overlap suggests that cognitive processes during language resolution and the executive inhibitory processes share the same brain structure (see [20, 21] for reviews). However, it has been argued that this overlap was observed because of the use of a verbal executive control task. According to this view, processes supporting such a verbal conflict resolution would be language-specific and would rely on language-specific brain regions [22, 23]. Recently, Hsu, Jaeggi, and Novick [6] showed that the LIFG could be considered as a conflict resolution hub that coordinates with different networks depending on the task (see also [24]). More specifically, authors showed that the LIFG was recruited during language conflict as well as during resolution of proactive interference in working memory (i.e., interference of previously learned materials on acquisition of newer materials) and during executive control. Therefore, it seems that both domain-general and domain-specific processes interact during conflict resolution in language comprehension tasks supporting the view of an involvement of executive control processes during language processing.

Additional evidence for such an involvement comes from neuropsychological studies. Vuong and Martin [25] tested three patients (two with a LIFG lesion and one control patient without a LIFG lesion) on an attentional control tasks as well as in a language comprehension task. Attentional control tasks consisted of a Stroop task, a Picture-word interference task (i.e., patients had to name a picture while ignoring the written word), a modified Recent-negative task (i.e., patients saw series of three words followed by a probe and had to tell whether the probe was presented in the series), and a short-term memory task. In the language comprehension task, patients read sentences containing or not a lexical ambiguity and then had to name a word. Patients with a LIFG lesion exhibited lower attentional control performances and more difficulties to resolve these ambiguities. Authors interpreted this result as evidence for the role of attentional control in lexical ambiguity resolution. Novick, Kan, Trueswell and Thompson-Schill [26] also showed such a pattern with LIFG lesion patients during comprehension of syntactically ambiguous sentences. More specifically, patients presented difficulties to inhibit and revise their first interpretation of syntactically ambiguous sentences supporting the view of an involvement of inhibitory processes during difficult language comprehension situations. Interestingly, a similar pattern of results has been observed among children who are known to have lower cognitive control abilities due to the slow maturation of the frontal cortex [27]. Children around the age of five showed more difficulties to follow temporarily syntactically ambiguous instructions than adults with less abilities to revise and inhibit their first interpretation [28–30].

On a behavioral level, several studies showed correlations between executive control performances and language processing [31, 32] suggesting shared cognitive processes between both executive control and language comprehension. For example, Hussey and collaborators [33] showed an improvement in language processing after a cognitive control training through a N-back task. During this training task, participants saw letters one by one and had to update information in working memory in order to be able to decide whether the presented letter was the same as the one presented three letters before (i.e. 3-back). After a N-Back training session, participants were tested on a language processing task. Participants who performed a high-conflict training reread faster and more easily ambiguous sentences than participants who performed a low-conflict training (see also [34, 35] for similar studies). Thus, training evidence suggested a causal effect of domain-general executive control on language performances. Similarly, Rodd, Johnsrude, and Davis [36] reported results suggesting that ambiguous sentences comprehension relied on domain-general processes. More specifically, participants listened to semantically ambiguous (containing a homophone) and unambiguous sentences and conjointly performed a case-judgement task. Authors manipulated the sentence linguistic contexts in order to induce a priori disambiguation (from prior context), an immediate disambiguation (from the words following the homophone) or a delayed disambiguation (from the end of the sentence). They evaluated the interference effect on the synchronized case-judgment task (i.e., decide whether a presented letter is in upper or lower case) on each disambiguation condition (prior, immediate or delayed disambiguation). Results showed that participants were slower on the case-judgment task for the delayed disambiguation condition compared to other conditions, i.e. they exhibited an interference effect. This result suggests that ambiguous sentences required executive control resources during the syntactic reanalysis of ambiguous sentences.

Together, these behavioral studies emphasize the view of an involvement, in some circumstances, of different executive control processes during language comprehension. However, the majority of these studies used verbal materials to evaluate such an involvement of executive control processes during language comprehension. In other words, the tasks used to evaluate executive control were designed with verbal or visuo-verbal items. Thus cognitive processes involved during such tasks could be considered as specific to language rather than more domain general processes letting uncertainties around processes at work during language comprehension. In an eye tracking experiment, Brown-Schmidt [37] was able to predict participants’ language comprehension performances based on a verbal inhibitory assessment but failed to do so based on non-verbal inhibitory assessment. Such result could be used as an argument for a domain-specific view of language comprehension but the author rather explained it as a ceiling effect or an absence of conflict inhibition in the non-verbal inhibitory task. In our study, we used a non-verbal executive control task in order to evaluate domain-general processes involvement during language comprehension.

The present study aims at evaluating the possibility of shared cognitive mechanism during verbal and non-verbal tasks and therefore the implication of domain-general cognitive control during language processing. Based on the assumption that the interference between two tasks reflects the recruitment of a common cognitive resource [38], we hypothesized that a behavioral cost will be observed during dual-task processing involving verbal and non-verbal tasks. To test this claim, we designed a dual-task paradigm involving an auditory language comprehension task (sentence comprehension) and a non-verbal Flanker task. We manipulated sentence ambiguity and evaluated if the ambiguity effect (related with a more difficult language processing) modified behavioral performances in the non-verbal Flanker task. We assumed that ambiguous sentences induce a more difficult language processing compared to unambiguous sentences. In our study, ambiguity is operationalized by the use of homophones. In ambiguous sentences all the possible meaning of the homophone are activated while in the unambiguous sentences the context preceding the homophone constrained and reduced activation of non-pertinent meanings of the homophone (see [39, 40]). Consequently, we expected performances on the Flanker items to be impaired only when a simultaneous difficult language processing was performed.

## Materials and methods

This study has been preregistered on the Open Science Framework website before data collection. The project including protocol and material is available at this link: https://osf.io/65saf/?view_only=7995e9d41aab4d978cbf7f3a72cf24ac

### Participants

Eighty students from Université Grenoble Alpes participated in this study (Mean age = 20.8yo, *SD* = 3.4yo) and received course credits for their participation. All were right handed and native French speakers with a normal or corrected vision and without any hearing problems. A power calculation for mixed effect models was computed using PANGEA’s website (Power ANalysis for GEneral Anova designs, v0.2) (see parameters’ details in the preregistered supplementary material) before the beginning of the experiment in order to assess how many participants were needed. This power calculation revealed a needed sample of 80 participants. Each participant gave written consent before performing the experiment. This study has been approved by the local ethical committee (CERGA [Comité d’éthique pour la recherche, Grenoble Alpes], N°CER Grenoble Alpes-Avis-2020-03-10-02).

### Task

**Fig 1:**
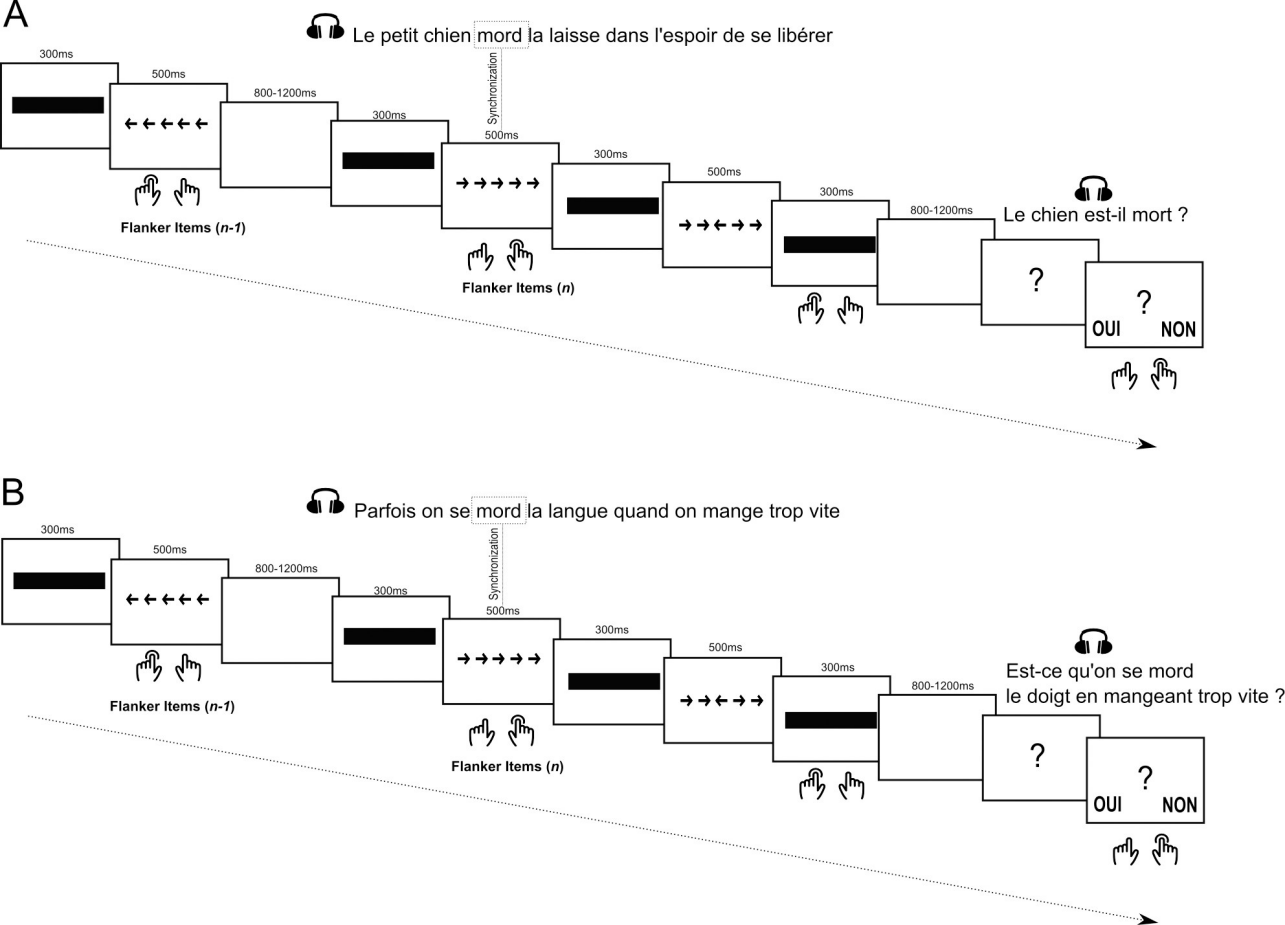
Dual-task paradigm involving a Flanker task and an auditory language comprehension task. Example of three consecutive Flanker items (with masks and inter-stimulus intervals) presented with (A) an ambiguous auditory sentence (“*Le petit chien* *mord* *la laisse dans l’espoir de se libérer*”; i.e., The little dog bites the leash hoping to free himself) and an auditory question by the end (“*Le chien est-il mort*?*”*; i.e., “Is the dog dead?”) or (B) a control auditory sentence (“*Parfois on se* *mord* *la language quand on mange trop vite”*; i.e., Sometimes we bite our tongue when we eat too fast) and an auditory question by the end (“*Est-ce qu’on se* *mord* *le doigt en mangeant trop vite*?*”*; i.e., “Do we bite our finger when we eat too fast?”). Participants responded with both hands for left or right and yes or no answers.

Participants were instructed to perform a dual-task paradigm involving: a Flanker task [41] and an auditory language comprehension task. More specifically, participants were instructed to listen to auditory sentences and to perform a Flanker task at the same time (see Fig 1). The Flanker task was designed to measure inhibition control and interference resistance by assessing ability to suppress predominant response. For the Flanker task, participants were instructed to decide whether a central arrow in a string of five arrows pointed to the right or to the left. For the auditory language comprehension task, participants were instructed to listen to the auditory delivered sentences and to be able to answer a yes/no question related to these sentences. See below for specificity of each task.

### Stimuli

#### Flanker items

The Flanker visual stimuli were strings of five arrows either all pointing to the same direction (left or right, these items are called congruent Flanker items), or with the central arrow pointing to the opposite direction of the other four arrows (incongruent Flanker items). A total of 520 trials were presented, half of them were congruent while the other half were incongruent. Strings of arrows were presented in the center of the screen (grey background) with a visual angle of 3.6 degrees (around 4 centimeters on the screen).

#### Auditory language comprehension stimuli

A set of 130 auditory French sentences were selected from the study of Millotte, René, Wales, and Christophe [42] and were included in the present study in three different experimental conditions: Ambiguous, Control and Fillers.

The Ambiguity condition included 20 pairs of temporary ambiguous sentences (mean duration = 2974ms; SD = 430ms). In these sentences, lexical ambiguity was manipulated by the presence of an auditory homophone word that is only disambiguated by the subsequent lexical context. For example, in the sentence “*Le petit chien* *mord* *la laisse dans l’espoir de se libérer*” (The little dog bites the leash hoping to free himself) the word “mord” (bites) presented auditorily can be interpreted either as the verb “mordre” (to bite) or as the adjective “mort” (dead) because of their similar pronunciation (/ m ɔ R /). In our example, the meaning of the homophone “*mord”* can be disambiguated with the following noun “*la laisse*” Consequently, in the Ambiguous condition, it is the following context that disambiguates the homophone meaning.

The Control condition, also included 20 auditory sentences (mean duration = 3093ms; SD = 602ms). These control sentences contained the same homophones as the Ambiguous conditions but were unambiguous due to a different sentence context prior to the homophone. In particular, the beginning of the control sentence restricted possible meaning interpretations of the homophone to the correct one. For example, in the control sentence “*Parfois on se* *mord* *la langue quand on mange trop vite”* (Sometimes we bite our tongue when we eat too fast) the context preceding the homophone restrict the interpretation of the word “mord” (bites) only to that of the meaning of “mordre” (to bite) and not to the one of “mort” (dead). Each homophone was heard four times: twice for each of its meaning (once in an ambiguous context and once in a control context) (see Table 1). Importantly, in the original study of Millotte et al. (2008) from which we used the stimuli, ambiguous sentences were shown to induce an ambiguity effect with slower response times compared to control sentences in a language task.

**Table 1:** Example of the four experimental conditions with a similar homophone presentation (the homophone word is in bold and underlined in the sentence).

Sentences stimuli	Sentence type	Flanker congruency
Parfois on se **mord** la langue quand on mange trop vite.	Control	Congruent
*Sometimes we bite our tongue when we eat too fast*.		
Le petit chien **mord** la laisse dans l’espoir de se libérer.	Ambiguous	Congruent
*The little dog bites the leash hoping to free himself*.		
Maintenant qu’il est **mort**, les batailles d’héritage vont commencer.	Control	Incongruent
*Now that he’s dead*, *the legacy battles will begin*.		
Le petit chien **mort** sera enterré demain par ses maîtres.	Ambiguous	Incongruent
*The dead little dog will be buried tomorrow by its owners*.		

The Filler condition included 50 auditory sentences (mean duration = 2903ms; SD = 599ms) with similar syntactic structure as control and ambiguous sentences. This condition was included in order to make sure participants do not guess experimental manipulation and hypotheses.

All the sentences used presented a minimally informative prosodic pronunciation (see [42]), in order to reduce the effect of prosody on ambiguity resolution and meaning selection. For each sentence, a yes/no comprehension question was recorded by a male speaker and presented at the end of the trial. Participants had 2500ms to respond after the end of the question.

All stimuli were presented using E-Prime software (v2.0.10.353, E-Prime Psychology Software Tools Inc., Pittsburgh, USA). Auditory stimuli were presented in a headphone with a comfortable sound level set by participants and manual responses were made on a keyboard. Participants gave their manual responses with both hands, right hand for right Flanker arrow direction and *no* answer to questions and left hand for left Flanker arrow direction and *yes* answer to questions.

### Procedure

Participants were instructed to listen to sentences and at the same time respond to Flanker items. They had to tell whether the central arrow of the Flanker items pointed to the left or the right. Sentences and Flanker items were synchronized in such a way that some Flanker items started at the same time as a homophone was presented in a sentence. This manipulation was designed to involve the lexico-semantic selection at the same time as the Flanker item processing (Fig 1). For filler sentences, the Flanker item appeared at the same time as a random word in the sentence. Before each sentence, two Flanker items were presented without any auditory presentation.

Each trial was made of four Flanker items and one sentence. The trials started with two Flanker items presented in silence. Flanker items started with a central rectangle (also used as a visual mask) presented for 300ms followed by the string of arrows presented for 500ms. Time between two Flanker items varied between 800ms and 1200ms in order to avoid participants to anticipate stimulus presentation and rhythmic responses. The third Flanker item was the one synchronized with the homophone for Ambiguous and Control condition or with a random word for the Filler condition. The fourth Flanker item appeared either by the end of the sentence or after the sentence depending on the sentence duration. Inter-trial intervals corresponded to a random duration between 800 ms and 1200 ms like between all Flanker items. Sentence type (Ambiguous, Control) and Flanker congruency (Congruent and Incongruent) were counterbalanced in order to obtain 20 trials for each condition crossing (see Table 1). Overall, participants performed 130 trials for a total of 130 sentences (40 Ambiguous, 40 Control, and 50 Fillers) and 520 Flanker items (260 Congruent and 260 Incongruent).

Finally, in order to make sure participants were involved in the two tasks, an auditory yes/no comprehension question was auditorily presented at the end of the trial. These comprehension verification questions were included in 75% of trials. Participants responded to Flanker items and to the question with two keyboard buttons. A short training session was proposed for the Flanker task; including 16 Flanker items (eight congruent and eight incongruent items). Reaction times and accuracy on all of the Flanker items and language comprehension questions were recorded.

### Data analyses

Statistical analyses were conducted using R version 4.0.3 (R Core Team, 2018), and lme4 [43] and lmerTest [44] packages.

#### Verification analyses

Before performing statistical analysis assessing the study hypothesis, we performed verification analysis evaluating: 1) accuracy of each task and 2) the dual-task cost. In order to be sure that participants performed the language task, we checked participants’ performances on the comprehension task by the evaluation of the behavioral responses to the comprehension questions. Before the experiment, we set a threshold for each task below which we would consider participants as not sufficiently involved in the tasks and exclude them from the statistical analyses (see pre-registered Method section of the project on OSF). These thresholds were set to 75% and 80% of correct answers for the comprehension task and the Flanker task, respectively. Then, we verified that listening to sentences during Flanker items interfered with reaction times to Flanker items. To do so, we compared reaction times of Flanker items performed while listening to a sentence (i.e., the Flanker synchronized with the homophone, we called here this condition: Flanker-in-sentence condition) and those performed in silence (we called here this condition: Flanker-in-silence condition) in a mixed effect model.

#### Confirmatory (preregistered) analyses

The main goal of this study was to evaluate whether common cognitive mechanisms are shared during difficult verbal and non-verbal tasks. More specifically, we evaluated the impact of difficult language processing, as manipulated by ambiguous sentences during auditory sentences presentation, on the behavioral performances for a non-verbal conflict resolution induced by a Flanker task. All these analyses were focused on the performances (reaction times and errors) obtained on Flanker items performed while listening to a sentence (i.e., the Flanker synchronized with the homophone).

Regarding our hypothesis, we expected a significant main effect of Flanker congruency, with slower reaction times for incongruent than for congruent Flanker items. We also expected a main effect of sentence type reflected by slower reaction times to Flanker items (both congruent and incongruent) when synchronized with ambiguous sentences than when synchronized with control sentences (i.e., unambiguous sentences). Additionally, we expected an interaction between sentence type and Flanker congruency. In other words, we expected a larger incongruency cost (i.e., larger difference between incongruent and congruent Flanker trials -in terms of both reaction time and accuracy-) during trials involving difficult language processing (i.e. ambiguous sentence condition) than during easy language processing (i.e., control sentences condition).

To statistically evaluate our hypotheses in terms of reaction times (RTs), we computed a mixed effect model on the RTs of the Flanker trials with, as fixed effects the following factors: sentence type (Ambiguous/Control), Flanker congruency (Congruent/Incongruent) and their interaction; and as random effects: the sentence’s intercept, the subject’s intercept, and the subject’s random slope for Flanker congruency. In order to reduce the weight of extreme data, reaction times were *log* transformed. Only correct responses were included in the RTs analyses. Regarding accuracy measure, a logistic mixed effect model was computed with as fixed effects the following factors: sentence type, Flanker congruency and their interaction; and as random effects: the stimuli’s intercept, the subject’s intercept, and the subject’s random slope for Flanker congruency. In both of these models, subject’s random slope for sentence type was remove in order to avoid a possible overfitting.

#### Exploratory (non-preregistered) analyses

Previous studies on the Flanker task have shown a *sequential congruency effect* [45, 46] suggesting an impact of the *n-1* congruency on the *n* performances. Consequently, is possible that we observe such a sequential effect in our paradigm between the *synchronized* Flanker items (*n* Flanker item) and the one that precedes it (*n-1* Flanker item) (see Fig 1). More specifically, it is possible that the congruency of the Flanker item before the *synchronized* Flanker item impacts the *synchronized* Flanker item performance. In order to consider a such possible effect, we included this factor in our model: specifically, we included the Flanker congruency of the *n-1* Flanker items. We computed a mixed effect model on the RTs of the Flanker items with, as fixed effects the following factors: sentence type (Ambiguous/Control), *n* Flanker congruency (Congruent/Incongruent), *n-1* Flanker congruency (Congruent/Incongruent), their interactions and the three-way interaction; and as random effects: the sentence’s intercept, the subject’s intercept, and the subject’s random slope for Flanker congruency. Moreover, in light of the sequential effect of the Flanker items, the inclusion of the *n-1* Flanker congruency in our model will allow to absorb a lot of variance and consequently provide a better understanding of the processes involved during the task.

Finally, we evaluated the possibility of a residual effect of the homophone processing. Indeed, in the present experiment, the control sentences were considered as unambiguous because the context preceding the homophone in the sentence driving lexical interpretation toward only one specific meaning of the homophone. Nevertheless, processing a homophone still involves different processing mechanisms than non-homophone words. For instances, in these control sentences, the context might indeed favor one interpretation of the homophone, but does not totally remove activation of other interpretations [47, 48]. Consequently, in order to exclude this residual effect, we compared ambiguous sentences to filler sentences, i.e., sentences without word activating multiple meanings. We included the filler sentences in the sentence type variable of the statistical model evaluating the interaction between Flanker congruency on *synchronized* Flanker item and sentence type (i.e., Ambiguous, Control and Filler sentences). Thus, we were able to compare ambiguous sentences to control ones but also to filler sentences and their interaction with Flanker congruency.

## Results

### Verification analyses

As previously suggested to ensure that all participants correctly perform the dual-task paradigm, we checked accuracy in both Flanker and language comprehension tasks. We showed that all participants performed correctly the language comprehension task with rates higher than 75% (mean percentage of correct answers = 92%; SD = 27%). Regarding performances to the Flanker items (mean percentage of correct responses = 94%; SD = 8%), three participants were below the threshold of 80% of correct answers and were removed from the analyses. In addition to these thresholds, we excluded participants deviating from more than two standard deviations from the overall mean reaction time. Thus, a total of six participants were removed from the analyses.

**Fig 2:**
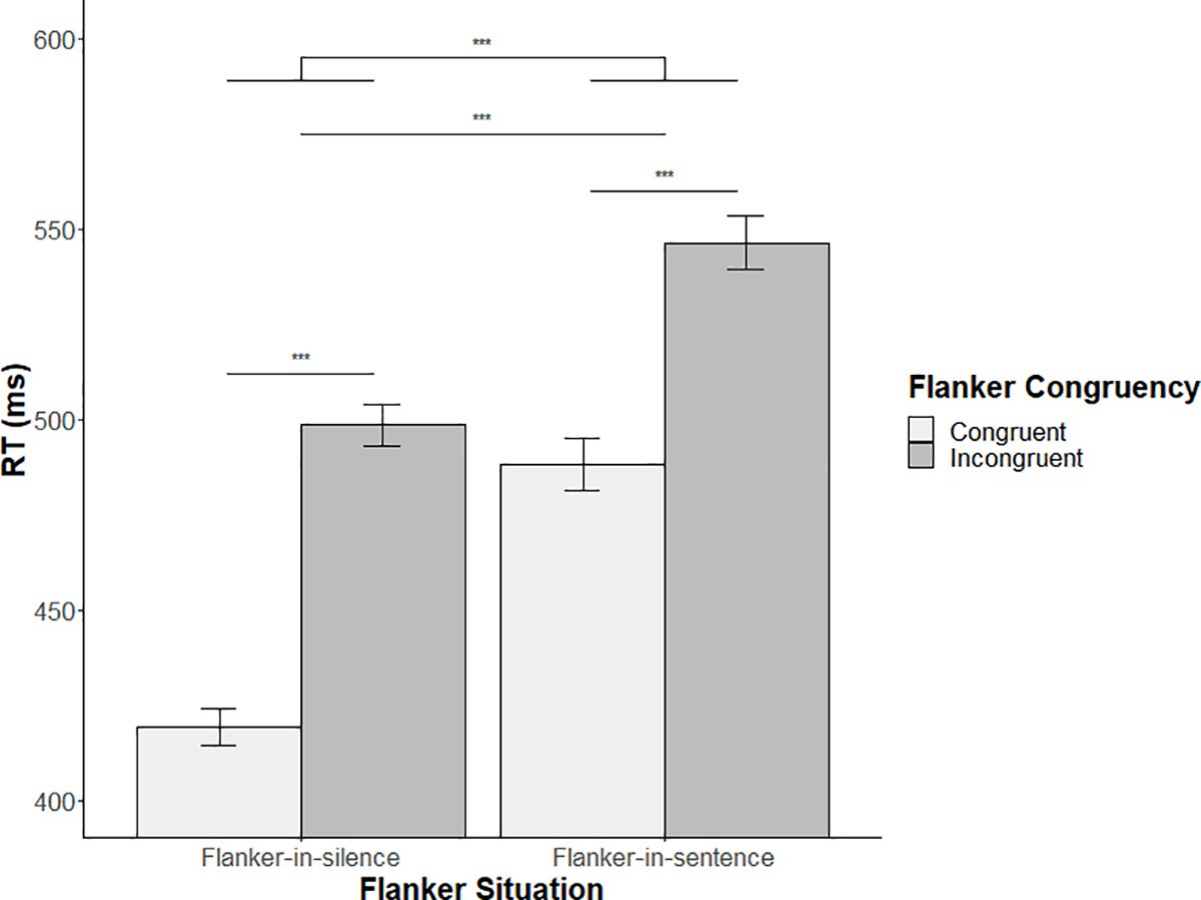
Reaction times for the Flanker items as a function of Flanker situation and Flanker congruency conditions. Note. ***p < .001.

We also evaluated the dual-task cost of participants by comparing participant’s performances to flanker items performed in silence (i.e., Flanker-in-silence condition) and Flanker items performed during sentence comprehension (i.e., Flanker-in-sentence condition). This analysis revealed a main effect of Flanker situation (i.e., Flanker-in-silence condition v.s. Flanker-in-sentence condition) (*t* = 24.46, β = 0.20, *SE* β = 0.008, *p* < .001). Participants were slower for Flanker–in-sentence items (mean RT = 516ms; SD = 128ms) than for Flanker-in-silence items (mean RT = 455ms; SD = 100ms). This main effect is observed for both congruent and incongruent Flanker conditions (Fig 2) and confirms a cognitive cost of doing two tasks at the same time in this paradigm. Results also show a significant main effect of Flanker congruency (*t* = 55.70, β = 0.17, *SE* β = 0.003, *p* < .001) with participants being slower for incongruent items (mean RT = 515ms; SD = 111ms) compared to congruent ones (mean RT = 441ms; SD = 104ms). A significant interaction between Flanker situation and Flanker congruency (*t* = -11.02, β = -0.06, *SE* β = 0.005, *p* < .001) was observed. More specifically, this interaction reveals a larger incongruency cost (i.e., slower reaction times to incongruent items than to congruent items) during the Flanker-in-silence (mean difference incongruent minus congruent = 80ms; SD = 22ms) condition than during the Flanker-in-sentence condition (mean difference incongruent minus congruent = 57ms; SD = 31ms).

### Confirmatory results

In terms of accuracy, analysis of Flanker congruency (Congruent/Incongruent) and sentence type (Ambiguous/Control) revealed a significant effect of Flanker congruency (*Z* = -5.21, β = -1.83, *SE* β = 0.35, *p* < .001, *d* = 1.53) showing that participants responded more correctly for congruent (M = 99%; SD = 12%) than for incongruent (M = 93%; SD = 26%) Flanker items. We observed no significant effect of sentence type (Z = 1.37, β = 0.97, *SE* β = 0.71, *p* = 0.17) as well as no significant interaction effect between Flanker congruency and sentence type (Z = -0.75, β = -0.29, *SE* β = 0.39, *p* = 0.46).

**Fig 3:**
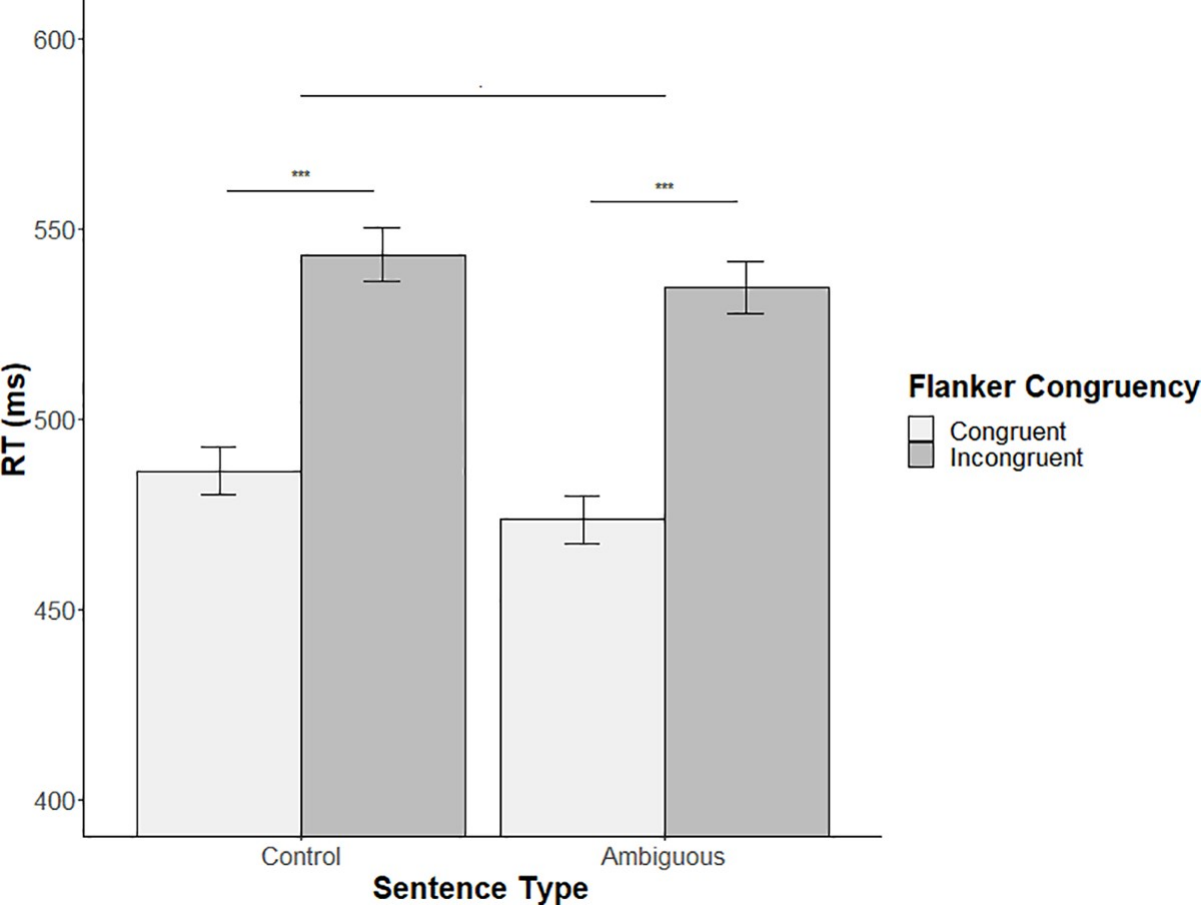
Mean reaction times for each experimental sentence type as a function of synchronized Flanker congruency. Note. p < .06.; ***p < .001.

In terms of reaction times, the computed model showed a significant main effect of Flanker congruency (*t* = 11.29, β = 0.11, *SE* β = 0.01, *p* < .001, *d* = 2.34). Participants were slower for the incongruent Flanker condition (mean RT = 539ms; SD = 119ms) than for the congruent condition (mean RT = 482ms; SD = 111ms). A marginal significant main effect of sentence type (*t* = -1.91, β = -0.39, *SE* β = 0.02, *p* = 0.059, *d* = 0.44) was observed in which participants seem to be faster to Flanker items while listening to ambiguous sentences (mean RT = 505ms; SD = 119ms) than while listening to control sentences (mean RT = 516ms; SD = 121ms). To evaluate the robustness of the sentence type marginal effect we also computed analyses from different statistical approaches: a classic repeated measure ANOVA with subjects as a random factor (S1 Table), a classic ANOVA with items as random factor (see S2 Table), and a Bayesian repeated measure ANOVA (see S3 and S4 Tables). All results of these additional analyses are presented in the Supporting information. Finally, no significant interaction between Flanker congruency and sentence type was observed (*t* = 0.92, β = 0.01, *SE* β = 0.01, *p* = 0.36). This absence of interaction effect prevents us from concluding on a modulated influence of the sentence type by Flanker congruency on Flanker items (Fig 3).

### Exploratory results

**Fig 4:**
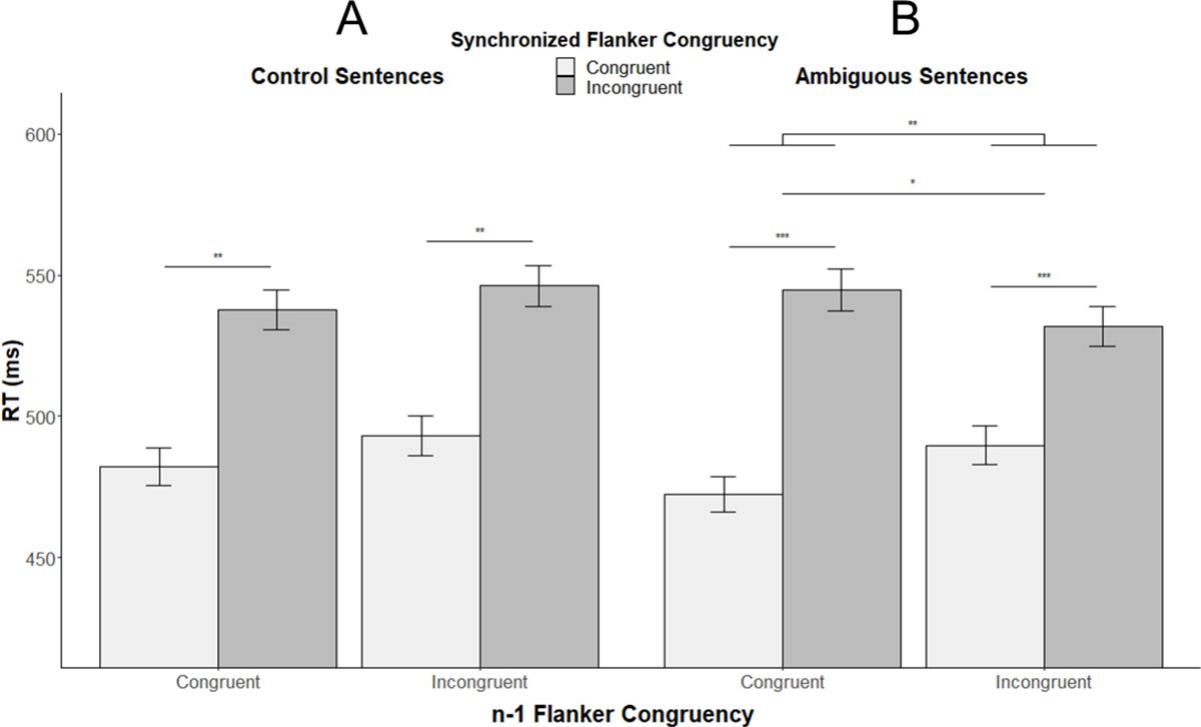
Mean reaction times to *synchronized* Flanker items as a function of *synchronized* and *n-1* Flanker congruency and sentence congruency. (A) shows a significant main effect of *synchronized* Flanker congruency for control sentences while (B) shows significant main effects of *n-1* and *synchronized* Flanker congruency as well as a significant interaction effect between the two variables for ambiguous sentences. *Note*. ^*^p < .*05*. **p < .*01*. ***p < .*001*.

The mixed model including *n-1* Flanker congruency, *synchronized* Flanker congruency and sentence type as fixed effects revealed a significant main effect of *synchronized* Flanker congruency (t = 3.73, β = 0.12, *SE* β = 0.03, *p* < .001, *d* = 0.84). As observed in the first model (confirmatory results), participants were faster for congruent Flanker items than for incongruent ones. Analysis revealed a marginally significant interaction effect between *synchronized* Flanker congruency and sentence type (t = 1.92, β = 0.08, *SE* β = 0.04, *p* = 0.058, *d* = 0.43), showing that the incongruency cost (reaction times to incongruent items minus reaction times to congruent items) was slightly smaller for Ambiguous sentences than for Control ones. Finally, we observed a marginally significant interaction between *n-1* Flanker congruency, *synchronized* Flanker congruency, and sentence type (t = -1.94, β = -0.5, *SE* β = 0.03, *p* = 0.056, *d* = 0.44) as shown in Fig 4. This marginal effect could suggest a different pattern of interaction between the *synchronized* Flanker congruency and sentence type as a function of the *n-1* Flanker congruency. This is why we also included in the analyses the interaction between *n-1* Flanker congruency and *synchronized* Flanker congruency for Ambiguous and Control sentences separately.

**Fig 5:**
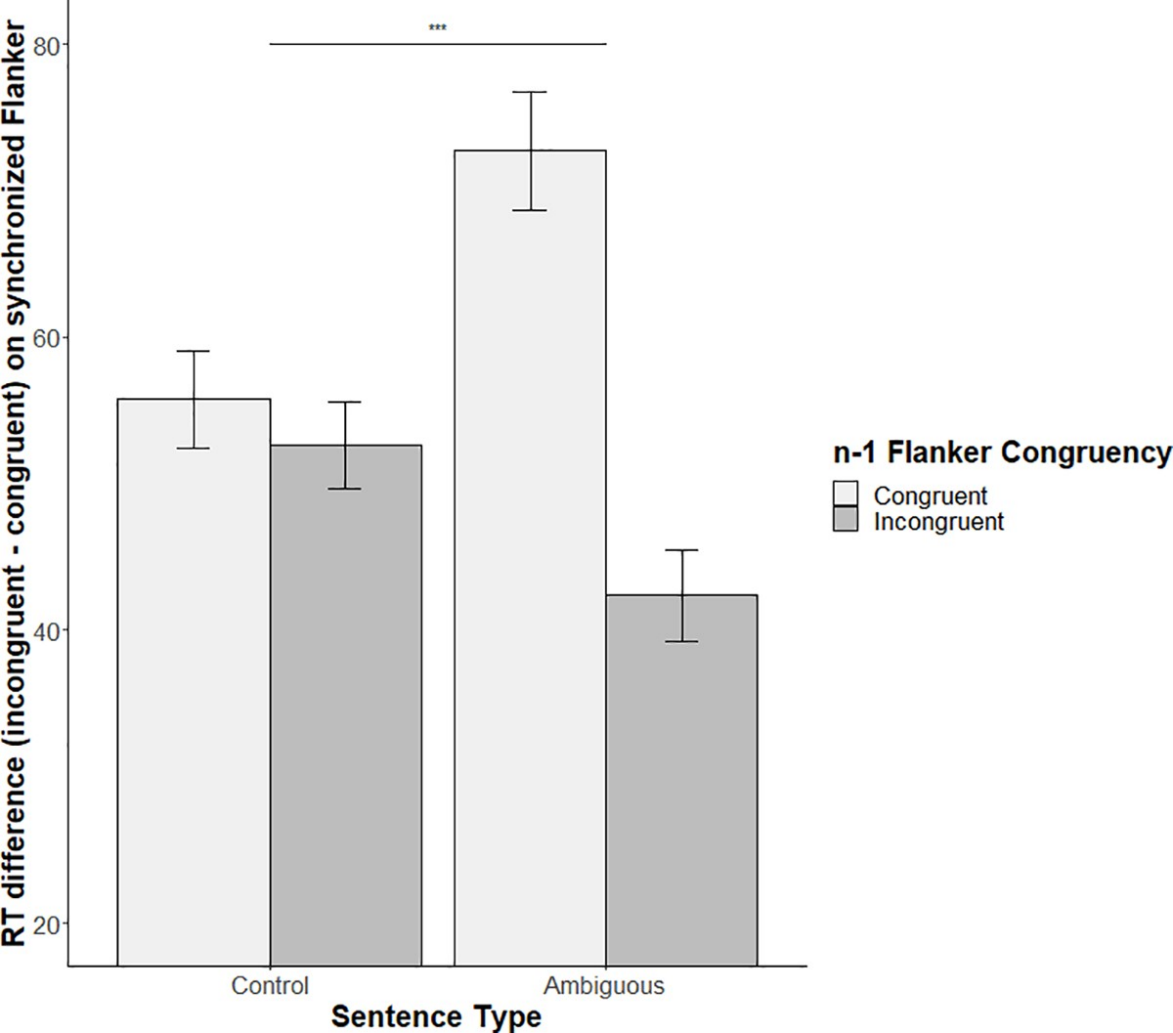
Mean reaction time difference between incongruent and congruent synchronized Flanker items as a function of sentence type and n-1 Flanker congruency.

Regarding ambiguous sentences, we observed a significant effect of *n-1* Flanker congruency (*t* = 2.69, β = 0.03, *SE* β = 0.01, *p* = .01, *d* = 0.86), suggesting an influence of *n-1* Flanker items on *synchronized* ones. More specifically, for ambiguous sentences, participants were slower on the synchronized Flanker item after an incongruent *n-1* Flanker item (mean RT = 521ms; SD = 122ms) than after a congruent one (mean RT = 490ms; SD = 117ms). We also observed a significant effect of *synchronized* Flanker congruency (*t* = 6.9, β = 0.2, *SE* β = 0.03, *p* < .001, *d* = 2.19), showing that participants were faster for congruent *synchronized* Flanker items (mean RT = 477ms; SD = 111ms) than for incongruent ones (mean RT = 535ms; SD = 123ms) when presented with ambiguous sentences. Finally, we observed a significant interaction effect between *n-1* and *synchronized* Flanker congruency presented with ambiguous sentences (*t* = -3.18, β = -0.06, *SE* β = 0.02, *p* = .003, *d* = 1.02). These results suggest that the incongruency cost for *synchronized* Flanker items is smaller (mean difference between incongruent and congruent items = 42ms; SD = 54ms) after incongruent *n-1* Flanker items than after congruent ones (mean difference between incongruent and congruent items = 73ms; SD = 70ms) when presented with ambiguous sentences (Figs 4B and 5). Regarding control sentences, we only observed a significant effect of *synchronized* Flanker congruency (*t* = 3.47, β = 0.12, *SE* β = 0.03, *p* = .001, *d* = 1.12) showing that participants were faster for congruent *synchronized* Flanker items (mean RT = 490ms; SD = 119ms) than for incongruent ones (mean RT = 540ms; SD = 121ms) without specific influence of *n-1* Flanker congruency (Figs 4A and 5).

The analysis including fillers sentences revealed a significant difference between ambiguous sentences and filler ones (*t* = 2.38, β = 0.06, *SE* β = 0.03, *p* < .05, *d* = 0.42). This effect suggests that participants were slower for Flanker items while listening to fillers sentences (mean RT = 526ms; SD = 125ms) than while listening to ambiguous sentences (mean RT = 505ms; SD = 119ms) (see S1 Fig). Moreover, when we took filler sentences into consideration in our model, the difference between ambiguous sentences and control ones did not reach significance level suggesting that this effect was small compared to the one of filler sentences.

## Discussion

The main goal of this preregistered study was to evaluate whether common cognitive control processes are shared during difficult verbal and non-verbal situations. Specifically, we evaluated the effect of homophone processing on a non-verbal conflict resolution task. We have for this purpose designed an experimental paradigm in which participants performed a non-verbal Flanker task synchronized or not with a homophone processing during an auditory sentence comprehension task. Participants were instructed to be involved in these two tasks at the same time (i.e., a dual-task paradigm). We expected the flanker task performances to be impaired only when a simultaneous difficult language processing was performed. This would be reflected by a performance cost during incongruent Flanker items for the more difficult language condition (ambiguous sentences) only.

Confirmatory results show a marginal effect *(p* = 0.059, *d* = 0.44) of sentence type on the Flanker task, in which participants seem to be faster to Flanker items while listening to an ambiguous sentence than a control one. It is important to highlight that given the paradigm used in the present study, in which the dependent variable measured was the reaction time to the *synchronized* Flanker items, this marginal main effect of sentence type can be interpreted as an influence of linguistic information processing on cognitive control processes at work during Flanker items. This marginal effect, even if it needs to be interpreted with caution, reveals a facilitatory effect in which difficult language condition facilitated the non-verbal inhibitory processes. It is important to note that this marginal effect was reversed with respect to our expectations. Indeed, we expected an interference effect, reflected by a Flanker item cost (i.e., a reaction time increase for the *synchronized* Flanker item) during difficult sentences (i.e., ambiguous sentence) due to shared cognitive resources. Such effect would have corroborated the surprisal account proposed by Hale [49] according to which cognitive control load corresponds to the amount of meaning possibilities rejected in a sentence (see also [50]). Instead, the present data showed that participants seemed to respond faster to Flanker items when presented with an ambiguous sentence than with a control one. This pattern of results is in line with the proposition of a cognitive control pre-activation effect during difficult language processing as previously suggested in the literature [13, 14, 51]. It is possible that the cognitive load induced by ambiguous sentences (multiple activated meanings), and therefore a more difficult language situation, pre-activates executive control processes needed for the Flanker item, leading to faster reaction times.

Our exploratory analysis including the filler sentences, provides evidences in line with this facilitatory effect. Indeed, participants exhibited slower reaction times during the filler sentences (i.e. without any cognitive load because only one meaning is activated) during sentence processing than during ambiguous sentences. Consequently, it seems that a greater language difficulty led to a facilitation of the concomitant inhibitory conflict resolution (see S1 Fig). Flanker congruency effect on the n-1 items also brings evidence toward a such facilitatory effect. Indeed, previous studies suggested that conflict detection initiates sustained cognitive control which attenuates the cost of subsequent conflict processing [45, 52]: like a pre-activation effect of the cognitive control process. This effect is in line with our exploratory analysis in which a three-way marginal interaction (*p* = 0.056, *d* = 0.44) between the *n-1* Flanker congruency, the *synchronized* Flanker congruency, and sentence type was observed. This marginal interaction is to be interpreted with caution. Nevertheless, it shows that the difference in reaction time between incongruent and congruent *synchronized* Flanker items is modulated both by *n-1* Flanker congruency and sentence type conditions. More specifically, the interaction effect between the congruency on the *n-1* and *n* Flanker (i.e. synchronized Flanker items) items is only observed during ambiguous sentences. Indeed, the incongruent *n-1* Flanker items seem to reduce the incongruency cost only during ambiguous sentences presentation. The fact that the interaction effect appears only for ambiguous sentences supports the hypothesis of a recruitment of executive control during difficult language processing. Furthermore, and even if this result need to be taken with caution because it is statistically marginal, it suggests that previous cognitive control pre-activation induces a facilitatory effect only when the following language processing is difficult.

This exploratory result is in line with findings reported by Hsu and Novick [14] in an EyeTracking paradigm. They observed a facilitatory effect of a previous increase in executive control load (through incongruent Stroop trials) on syntactic resolution trials. Recently, Thothathiri, Asaro, Hsu and Novick [13] used the same EyeTracking paradigm with active and passive sentences processing and also showed a facilitatory effect of previous high executive control load on the more difficult language condition (i.e., passive sentences). These studies show a direct relation between executive control processing arising from Stroop conflict and complex or difficult language conditions processing. It is important to highlight that these studies use executive control task in a verbal modality with the Stroop task, and this could be interpreted as the implication of a domain-specific conflict resolution mechanism. In our study, we used a non-verbal executive control task with the Flanker task in order to evaluate the possible implication of a domain-general mechanism during language processing. This way, our findings add evidence to the domain-general cognitive control account and suggest an involvement of non-verbal executive control during difficult language processing.

The present study presents several limitations. First, some results we are interpreting are marginally significant and would need to be replicated. Second, it is possible that the control sentences present a residual homophone effect (i.e., multiple meanings activated) as they contained the same homophone as those used in the ambiguous sentences. Even if the pervious homophone context is supposed to disambiguates and lead the interpretation toward the correct meaning [42, 53, 54], it is possible that a minimal activation of the other meanings remains. Consequently, the control sentences used might not be the most favorable condition for demonstrating the effect assessed in the present study. Another limitation can be related to the strength of the Flanker effect. Indeed, as previously mentioned, the Flanker effect is a strong behavioral effect that could mask other processes at work during this task. For this reason, it could be interesting to use a similar paradigm with another non-verbal executive task instead of the Flanker task in order to better identify the different executive control mechanisms that are at work during difficult language processing. In our paradigm, it would also be very interesting to synchronize the Flanker item on the disambiguation point or after the disambiguation to have a better idea of cognitive load throughout sentence processing.

Further research is needed to investigate which executive control processes are at work during language comprehension. Different executive functions could be involved during different steps or types of language processing. For example, revising a syntactic ambiguity might involve inhibition processes and working memory as one has to inhibit first interpretation of an ambiguous sentence when realizing we are misinterpreting it and maintain already processed linguistic information in order to be correctly interpret the sentence [36, 55]. The current study focused on non-verbal executive processes that might be at work during difficult language processing (multiple meanings activated). In a study with patients with aphasia, Hoffman, Rogers and Lambon Ralph [56] showed that selection between several semantic meanings of words rely on control processes. In fact, authors showed that patient with aphasia, who are known to have difficulties inhibiting semantics associations [57], exhibited a frequency effect on a synonym judgment test only when semantic diversity of words (i.e., how many meanings and semantic associations words can have) was taken into account. This study presents evidence for an involvement of inhibitory processes during lexical selection as more diverse words have more related meaning and more semantic associations that need to be inhibited in order to correctly perform the task. In a more recent study with two patient with aphasia, Nozari [58] was able to segregate activation and inhibition processes involved in meaning selection during language production (see also [59] for an interesting review on access deficits). Such a distinction between activation and inhibition during meaning selection might also appear in language comprehension and could be important to better rehabilitate patients with language deficits.

### Conclusion

In this study, we evaluated the influence of difficult language processing on a concomitant Flanker task. Results seemed to show a facilitatory effect of difficult language processing on Flanker processing. Thus, this study presents evidence for an involvement of non-verbal executive control processes during difficult language comprehension. Moreover, analysis considering *n-1* Flanker items suggests an interaction between cognitive processes at work during Flanker items and difficult language processing reflecting the complex interplay between domain-general and domain-specific language processing.

## Supporting information

S1 Fig Reaction times to Flanker items as a function of Flanker congruency and sentence type. Note. *p < .05. ***p < .001.
(TIF)

S1 Table Statistical results of the by-subjects repeated measure ANOVA. This analysis reveals a significant effect of sentence type; F(1,73) = 16,54, *p* < .025.
(TIF)

S2 Table Statistical results of the by-items ANOVA. This analysis reveals a significant effect of sentence type; F(1,76) = 8,39, *p* < .025.
(TIF)

S3 Table Statistical results of the Bayesian repeated measure ANOVA. This analysis reveals that the model includes both Flanker congruency and sentence type is the most probable with a bayes factor of 15,21.
(TIF)

S4 Table Analysis of the effects of the Bayesian ANOVA. This analysis shows that data are 133.3 times more probable under models that include the sentence type factor.
(TIF)
