# Supplementary figures and images for: Influence of homophone processing during auditory language comprehension on executive control processes: A dual-task paradigm
Source: PLoS One. 2021 Jul 15;16(7):e0254237. doi: 10.1371/journal.pone.0254237 (PMC8282032; doi:10.1371/journal.pone.0254237)

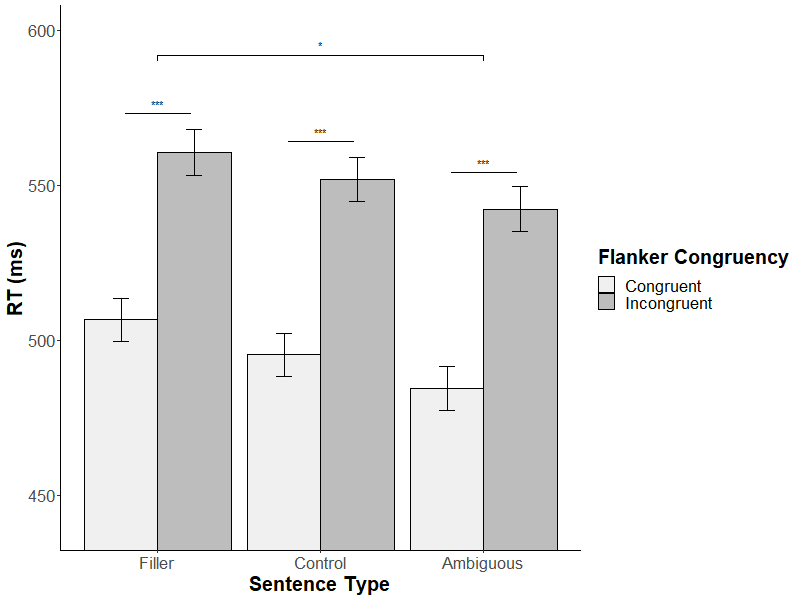

Supplement: S1 Fig — Note. *p < .05. ***p < .001. (TIF) [file pone.0254237.s001.tif]

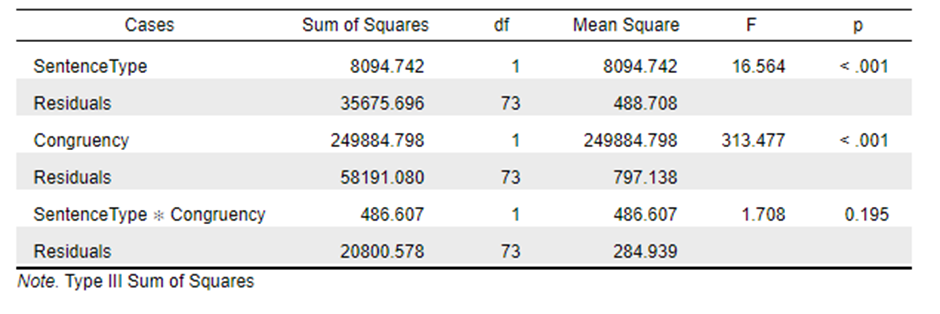

Supplement: S1 Table — This analysis reveals a significant effect of sentence type; F(1,73) = 16,54, p < .025. (TIF) [file pone.0254237.s002.tif]

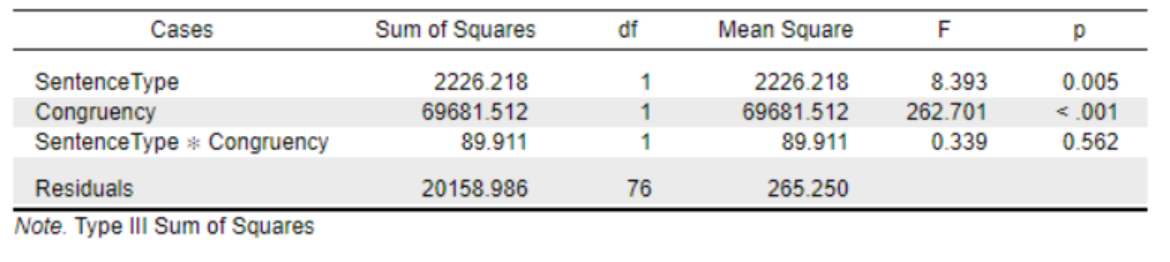

Supplement: S2 Table — This analysis reveals a significant effect of sentence type; F(1,76) = 8,39, p < .025. (TIF) [file pone.0254237.s003.tif]

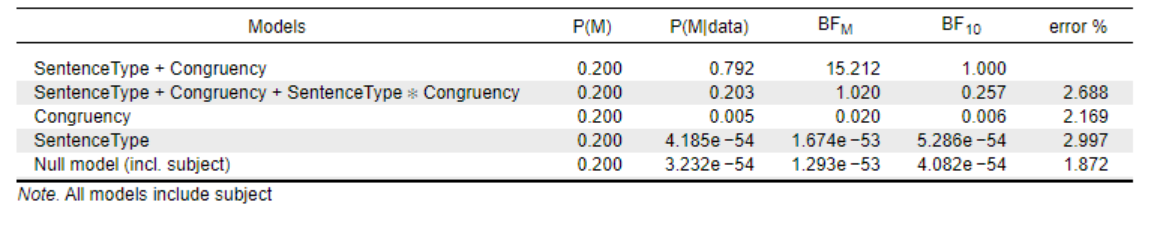

Supplement: S3 Table — This analysis reveals that the model includes both Flanker congruency and sentence type is the most probable with a bayes factor of 15,21. (TIF) [file pone.0254237.s004.tif]

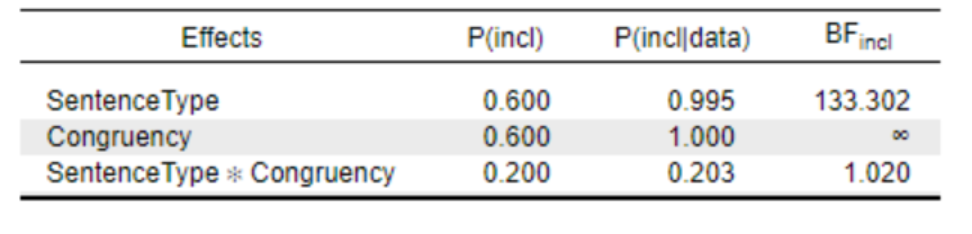

Supplement: S4 Table — This analysis shows that data are 133.3 times more probable under models that include the sentence type factor. (TIF) [file pone.0254237.s005.tif]
